# Different associations of premorbid intelligence *vs*. current cognition with BMI, insulin and diabetes in the homebound elderly

**DOI:** 10.15761/IMM.1000202

**Published:** 2016-02-19

**Authors:** Mkaya Mwamburi, Wei Qiao Qiu

**Affiliations:** 1 Department of Public Health and Family Medicine, Tufts University, Boston, USA; 2 Departments of Psychiatry, Boston University School of Medicine, Boston, US A; 3 Pharmacology, Boston University School of Medicine, Boston, USA; 4 Alzheimer’s Disease Center, Boston University School of Medicine, Boston, USA

**Keywords:** diabetes, homebound elderly, verbal IQ, cognitive impairment

## Abstract

Premorbid intelligence does not decline through life even at the early stages of Alzheimer’s disease (AD). However, other cognitive measures such as Mini Mental State Examination (MMSE) decline with aging and severely with dementia. In this study, we examine the associations of premorbid intelligence vs. current cognition with body mass index (BMI), insulin and diabetes in elderly adults. Using a cross-sectional, population-based study, we assessed BMI, plasma insulin and the evidence of diabetes in homebound elders. The North American Adult Reading Test (NAART) and MMSE were conducted. Associations were assessed by T-test, linear correlation and multivariate regression analysis. Subjects were divided into four subgroups: 1) BMI <25; 2) 25 < BMI <30; 3) 30 < BMI <35 and 4) BMI >35. Lower verbal IQ, assessed by NAART, was associated with higher BMI (β=−0.28; P<0.01), elevated insulin (β= −0.02, P=0.02), and diabetes (β=− 3.18, P<0.01). Multivariate regression analyses showed that all three clinical conditions – obesity, hyperinsulinaemia and diabetes – were associated with lower premorbid intelligence assessed by verbal IQ, but only diabetes was associated with current cognitive impairment assessed by MMSE. These relationships persisted after adjustment for education. Premorbid intelligence is associated with diabetes precursors – obesity and high insulin – and diabetes itself, but cognitive impairment is related to diabetes only. Understanding the mechanisms that link verbal IQ to diabetes precursors might suggest targeted interventions for the prevention of diabetes and cognitive decline caused by diabetes.

## Introduction

Type 2 diabetes is associated with cognitive impairment but the relationship of its precursors, obesity and elevated insulin, to cognition has been less studied. Several studies have shown that type 2 diabetes is associated with cognitive impairment [1–6]. The underlying pathology of cognitive impairment in elderly with diabetes could be Alzheimer’s disease (AD) or vascular dementia (VaD) [7,8]. Insulin concentration post glucose loading has also been associated with cognitive impairment as well as AD among the subjects who do not carry ApoE4 allele [4,9,10]. Some study has demonstrated that obesity is associated with cognitive deficit in men, but this relationship has not been observed in women [13]. Both obesity and insulin resistance syndrome are the major risk factors for developing type 2 diabetes. The course from obesity to insulin resistance syndrome, to type type 2 diabetes is chronic, and many believe that treating obesity could prevent or delay the onset of type 2 diabetes [11]. Therefore, understanding the association of cognitive deficits with each clinical condition – obesity, insulin resistance syndrome, type 2 diabetes – may help in preventive strategies against cognitive decline in the elderly with type 2 diabetes.

Lower general intelligence may be a risk factor for both the onset of dementia and the rate of cognitive decline in aging through the mechanism of cerebral reserve [12,14]. Premorbid intelligence is mainly a heritable trait, and can be affected by environmental factors or diet in early childhood. The North American Adult Reading Test (NAART) is a measure of verbal intelligence, which is resistant to age and dementia, and is therefore considered to be a measure of premorbid intelligence [15]. On the other hand, the Mini-Mental State Exam (MMSE), which declines with age and demented diseases such as AD, is a measure of current cognition [16]. Although the NAART and the MMSE represent different brain functions and have different relationships with regard to aging and demented diseases, the two are correlated with each other, and also with education [17]. Several studies have shown an association between type 2 diabetes and cognitive decline, yet no study we know of has shown relationships between premorbid intelligence and obesity, insulin resistance syndrome and type 2 diabetes in elderly.

The purpose of this study is to characterize the three clinical stages of type 2 diabetes development – obesity, insulin resistance syndrome and diabetes – with regard to both baseline intelligence and current cognition. Using a simple but well-validated test, the NAART, to evaluate verbal IQ and the MMSE to measure current cognitive condition, we examined these relationships in a homebound elderly population.

## Materials and methods

### Study population and recruitment

The subjects were from the first 301 participants of an ongoing, population-based study of homebound elderly, *Nutrition and Memory in Elders (NAME study)*. The sample frame consisted of clients of three homecare agencies for the city of Boston. Anyone receiving homecare services is registered with one of these agencies if he/she lives in the city of Boston, has an annual income < $18,890 and needs homecare service. All homebound elders aged 60 and older at each of the three agencies were invited to participate in the study. To be eligible to be enrolled, the participants must speak English, be physically able to participate in the study home visits, and have sufficient vision and hearing to read and hear the content of the neuropsychological tests. All enrolled subjects gave informed consent. The protocol and consent form were approved by the Institutional Review Board of Tufts University New England Medical Center.

### Data collection

Each subject participated in three home visits. The neuropsychological battery was accomplished during the first home visit. At the second home visit, blood was drawn before breakfast under fasting conditions for at least 7 hours. During the third home visit, body mass index (BMI) was measured, and information on health condition and medication use was collected. Neuropsychological tests were given without prior knowledge of whether subjects had diabetes.

### Measurements

#### BMI and Diabetes

Weight and stature were measured twice using standardized instruments, and the average of two measurements was used to calculate BMI (kg/m^2^). Diabetes was defined as the use of anti-diabetic medication or fasting glucose greater than 126 mg/dl, parameters widely used by the different population-based studies [7,18]. Subjects were asked to show all medications they were taking, and research assistants documented the medication names according to the labels. Glucose concentrations were measured using the glucose hexokinase method, and serum insulin was measured by routine radioimmunoassay.

#### Verbal IQ and Cognition

Research assistants, who were trained by a board certified neuropsychologist, administered the cognitive tests during the first home visit. Two tests are used in this analysis: 1) the North American Adult Reading Test (NAART) [15,19] {; and 2) the Mini Mental State Examination (MMSE). The NAART includes 61 words, and each incorrectly pronounced word counts as one error. Verbal IQ is estimated by the equation: VIQ=128.7−0.89 × (number of word pronunciation errors). The MMSE contains 6 categories to measure orientation, registration, memory, attention/concentration, language and copy. The subjects with MMSE ≤ 10 or NAART <75 were not eligible to continue in the study. Education levels were divided into three categories for this analysis: 1) 0–8; 2) 9–12; 3) >12 school years.

### Statistical analysis

Statistical analysis was performed using PC-SAS (version 8.1). Demographic characterization, NAART and MMSE scores among the four subgroups with different BMIs were analyzed using analysis of variance (ANOVA). T-tests were used to compare the results of two subgroups based on BMI, insulin and diabetes status. P values for significance were calculated.

Spearman linear correlation and simple univariate regression were used to evaluate the relationship between each measure with the NAART and MMSE scores. Distributions of relationships between NAART, MMSE and age were plotted. Multivariate regression analysis was performed to evaluate the effect of potential confounding variables with the NAART and the MMSE scores as outcomes. Three education groups were used to adjust for education in the regression models.

## Results

### Study population

The first 301 recruited subjects (79% participation rate) were used for the analysis. The average age of this study population was 76.3 years old, 77% were female, and 69% lived alone. The population was multiethnic with 60% white, 38% African American and 2% other ethnicities. BMI data was obtained from 287 subjects. The mean ± SD of BMI was 31.7 ± 8.5 kg/m^2^. Blood measurements were missing for 22 (7.3%), due to logistical or technical blood draw problems, leaving a total of 278 subjects with plasma glucose and insulin measurements. Two hundred and ninety-one subjects answered the diabetes questionnaire, and 117 (40%) had diabetes. The NAART and MMSE data were complete for all subjects.

### Demographic and metabolic data across BMI status

Subjects were divided into four groups based on their BMI: 1) BMI ≤ 25 (N=67); 2) 25 < BMI ≤ 30 (overweight N=70); 3) 30 < BMI ≤ 35 (obese N=67); 4) BMI >35 (extremely obese N=83) (Table 1). Table 1 shows the comparison of descriptive status among the four subgroups in the homebound population. As mean BMI increase, mean age decreased in each subgroup (P=<0.0001), indicating that older age was correlated with lower body mass.

As mean BMI increased, mean plasma insulin concentration increased in each subgroup (Table 1, P=0.0001). Insulin resistance syndrome, which includes high BMI and elevated insulin concentrations, was prevalent in the obesity subgroups (30 < BMI ≤ 35 and BMI >35). Higher prevalence of diabetes was consistently observed with higher BMI (12%, 30%, 46% and 60%, P<0.0001). 78% of subjects with diabetes had diabetes for less than 20 years. The majority of subjects with diabetes were either on oral diabetic medication(s) only or not on any medication treatment for diabetes (~70%), indicating that a majority were suffering from type 2 diabetes.

### Verbal IQ and cognitive data across BMI status

**Figure 1:**
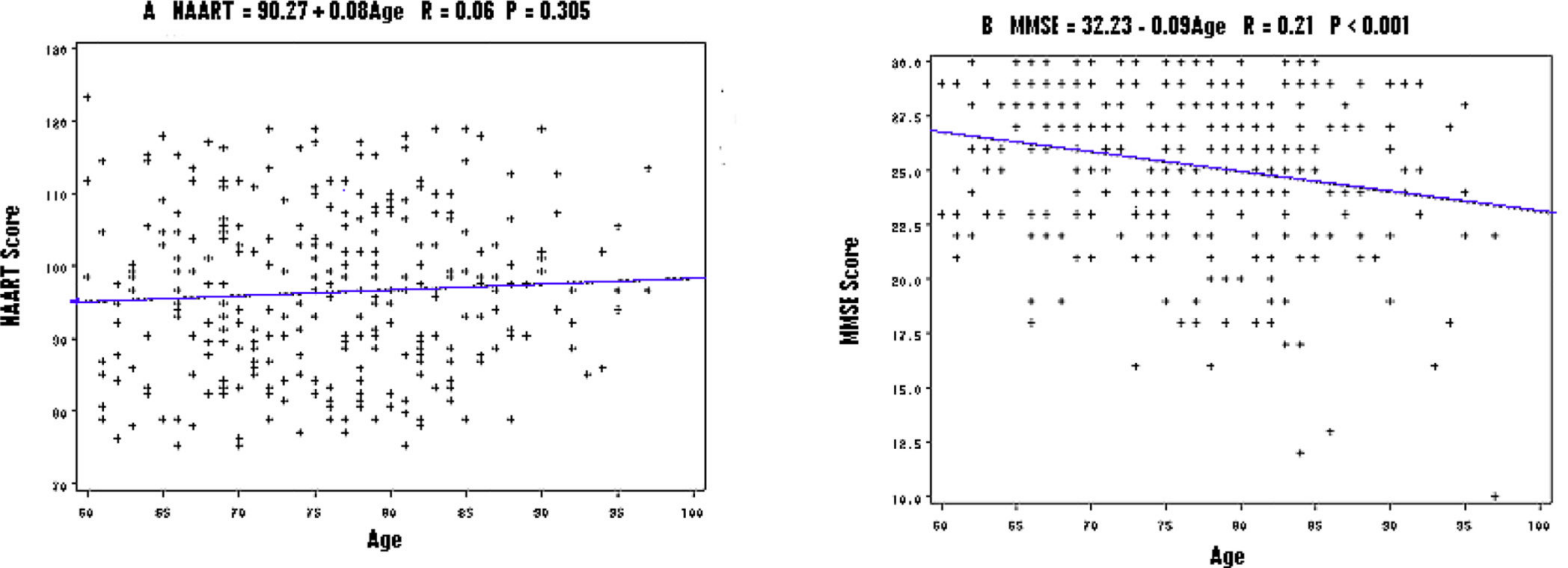
Scattered plots of age and verbal IQ (Figure 1A) vs. age and current cognition (Figure 1B), NAART: the North American Adult Reading Test, MMSE: the Mini-Mental State Examination, formulations for the relationships, r for correlation coefficient, P for statistical significance

NAART was not correlated with age (R=0.06, P=0.305) (Figure 1A) while MMSE was conversely correlated with age (R=0.21, P < 0.001) (Figure 1B) in the homebound elderly. These data illustrate that NAART measures baseline premorbid intelligence, which does not change with aging, but MMSE measures current cognitive function, which changes, as people age or become demented.

The mean ± SD of NAART for the lowest BMI subgroup was 101 ± 11 (Table 2). This was significantly greater than the scores for the higher BMI subgroups (98 + 12; 96 ± 11 and 93 ± 11, P=0.0003). These data were consistent with educational levels in the BMI subgroups (Table 1). The highest BMI subgroup was most likely to have low education (< 8 years) as compared to the lower BMI subgroups (as BMI decreased, 39% vs. 24%, 24% and 13%). In parallel, the lowest BMI subgroup was most likely to have high education (> 12 years) as compared to the subgroups with higher BMI of education: 30% vs. 27%, 19% and 24%) (Table 1). In contrast, current cognitive condition, assessed by MMSE score, was not different among the four BMI subgroups (Table 2) (P=0.9140).

### The converse relationship of verbal IQ with BMI, insulin concentration and diabetes

Lower NAART scores were associated with higher BMI measurements with β=−0.28 (P<0.001) in the univariate regression analysis (Table 3). Lower NAART scores were also associated with higher fasting insulin (β=− 0.02 and P=0.02) and with diabetes ((β=− 3.81 and P<0.01). The multivariate regression analysis showed that BMI was associated with verbal IQ (β=−0.20, SE=0.09, P=0.03) while age was not (β=−0.001, SE=0.09, P=0.91) (Table 4). When diabetes was not included in the regression model, β estimate (SE) for BMI and NAART became – 0.25 (0.09) (P<0.01). When BMI was not included in the regression analysis, both insulin and diabetes showed significantly negative relationships with NAART (Table 4), suggesting that BMI, insulin and diabetes influenced the outcome of verbal IQ through a probable common pathway.

These relationships persisted even after adjustment for education (Table 4). In addition, NAART scores and BMI measurements were plotted and found to have a bilinear phenomenon: a large slope among the elderly with BMI ≤ 40 kg/m^2^ and an almost flat slope among the elderly with BMI > 40 kg/m^2^ (data not shown). BMI*BMI was therefore introduced into the regression model (Table 4) to include this bilinear relationship. This resulted in a better fit for the relationship between BMI and verbal IQ in this population (β=−1.29, SE=0.40, P<0.01).

**Figure 2:**
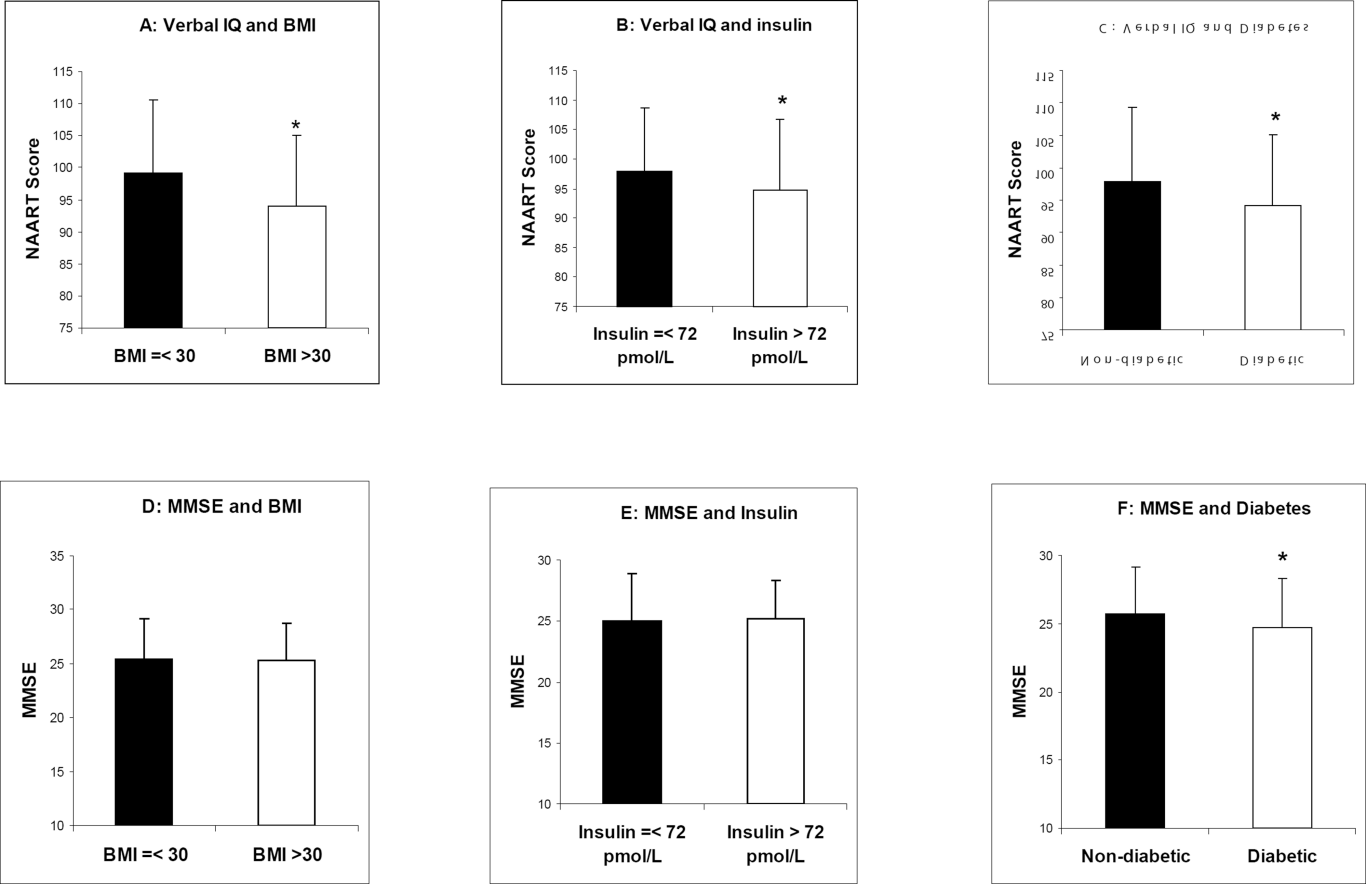
The relationships of verbal IQ with BMI (A), insulin (B) and diabetes (C) vs. current cognition with BMI (D), insulin (E) and diabetes (F) are illustrated. BMI is divided according to the cut-off point for obesity (30 kg/m^2^). Insulin is divided according the median concentration in the population. * P<0.05.

To confirm these relationships, the subjects were divided into two subgroups: BMI ≤ 30 kg/m^2^ and BMI >30 kg/m^2^. The obesity group (BMI > 30 kg/m^2^) had a significantly lower NAART score than the non-obesity group (BMI ≤ 30 kg/m^2^) (mean ± SD: 94.10 ± 10.10 *vs.* 99.24 ± 10.97, P < 0.001) (Figure 2A). High insulin was defined by the median >72 pmol/l in this population. Consistently, those with high insulin had a lower average NAART score compared to those with low insulin concentrations (mean ± SD: 94.65 ± 12.13 *vs*. 98.21 ± 10.52, P=0.010) (Figure 2B). The average NAART score of those with diabetes was lower than those without diabetes (mean ± SD: 94.10 ± 11.06 *vs*. 97.91 ± 11.47, P=0.006) (Figure 2C). All of these results (Figure 2) were consistent with the data from the univariable regression analysis shown in Table 3.

### The relationship of current cognition with BMI, insulin and diabetes

Unlike verbal IQ, current cognitive status, measured by MMSE, was associated with diabetes only, not with BMI or insulin (Table 3). In the multivariate regression analysis (Table 4), only diabetes (β=−1.41, SE=0.46, P<0.01) and age (β=−0.11, SE=0.03, P<0.01) were associated with MMSE. These relationships held with further adjustment for education (Table 4).

There was no difference in MMSE scores by obesity group (BMI > *vs.* ≤ 30 kg/m^2^) (Figure 2D). The high vs. low insulin groups also did not show statistical differences in MMSE mean score (Figure 2E). However, those with diabetes, had significantly lower MMSE scores than those without diabetes (mean ± SD: 24.68 ± 3.63 *vs.* 25.72 ± 3.51, P=0.016) (Figure 2F).

## Discussion

This is the first community-based study we know of to examine premorbid intelligence with regard to obesity, elevated insulin and diabetes in elderly. Our study describes the different relationships between verbal IQ vs. current cognition and three clinical conditions – obesity, insulin resistance syndrome and diabetes – in homebound elders.

General intelligence is resistant to aging, but current cognition declines with aging.

Verbal IQ, as measured by NAART score, was static across age while current cognition, as measured by MMSE score, declined with age in the homebound elderly (Figure 1). General intelligence is a heritable trait, and both genetic and environmental factors during early development influence intelligence levels that individuals reach by adulthood. While low intelligence might be a risk factor for both the onset of dementia and the rate of cognitive decline in aging, its measurement is static throughout life and resistant to both aging and the onset of dementia [15]. In contrast, cognition develops after birth to adulthood and declines during the aging process, deteriorating severely after the onset of dementia. Although current cognition is highly correlated with intelligence [16], the two measure different functions in the brain, and present differently in the aging process and in demented diseases.

Obesity is related to verbal IQ but not to current cognition in the elderly. It is noteworthy that, as BMI increased, the verbal IQ scores decreased (Table 2 and 3; Figure 2A). High BMI was also associated with low education in these elders (Table 1). A few cross-sectional studies have shown the inverse association of high BMI (> 31 kg/m^2^) with both low intelligence-test scores and low education levels in children and young adults [20–23]. One longitudinal study demonstrated that both intelligence and education were inversely associated with obesity, but only lower education predicted the risk of remaining obese from juvenile to adulthood [24]. In contrast, we found that current cognition, as measured by MMSE, was not associated with BMI in this population (Table 2 and 3; Figures 2D).

Elevated insulin is associated with lower verbal IQ but not with current cognition. Obesity is associated with increased insulin levels, and induces insulin resistance syndrome. No published study we know of has shown the relationship between fasting insulin and intelligence. We found that the fasting insulin concentration and diabetes prevalence rose as BMI increased (Table 1), and that insulin concentrations were inversely associated with verbal IQ score (Table 3; Figure 2B). The significant relationship between elevated insulin and lower verbal IQ may mediate the inverse relationship between BMI and verbal IQ.

Although two studies have shown that cognitive impairment was associated with elevated plasma insulin level two hours post glucose loading (oral glucose tolerance test) [25,26], we did not see an association between fasting serum insulin and MMSE score. Insulin concentration post glucose loading is physiologically different from the fasting insulin concentration used in this study, which may explain the different relationships to cognition.

Diabetes is associated with both verbal IQ and current cognition. Obesity and insulin resistance syndrome increase the risk of developing type 2 diabetes. In this study diabetes was associated with both lower verbal IQ scores and lower cognitive status (Figure 2C and 2F). Several studies have shown that type 1 diabetes in children is related to lower intelligence scores, but the relationship between premorbid intelligence and type 2 diabetes is unknown. In addition, multiple studies have shown that type 2 diabetes is associated with cognitive decline in the elderly, which is supported by our study.

In implications of the study, obesity, insulin resistance and diabetes were all related to lower verbal IQ through a possible common pathway (Table 4). On the other hand, only diabetes and age, but not obesity or fasting insulin, were associated with lower cognition, MMSE, (Table 4, Figures 2C and 2F).

Since mildly mentally retarded people have higher rates of obesity [27,28], it is possible that there is a common genetic origin for both IQ and BMI. On the other hand, one study shows that Prader-Willi patients who were diagnosed in infancy and received early behavioral treatment had lower BMI measurements and higher IQ scores than patients who were diagnosed and treated in the adulthood [29]. This suggests that both genetic and environmental factors determine premorbid intelligence levels.

Our study clearly shows that obesity was closely related to lower intelligence, which was also correlated with low educational status. It is, therefore, possible that low intelligence contributes to unhealthy dietary patterns, which lead to obesity and hyperinsulinaemia, then to type 2 diabetes. One study has shown that lower educational status raises the risk of remaining obese from juvenile to adulthood [24]. More efforts are needed to target young people with lower IQ scores or educational levels in order to keep them in a healthy BMI range to prevent the onset of type 2 diabetes and consequent cognitive decline as they age. Because premorbid intelligence is correlated with cognition [17], another area for future study might be to examine cognitive impairment in type 2 diabetics while accounting for the effects of premorbid intelligence.

In limitations of the study, the cross-sectional design of this study does not enable us to conclude the causal relationships of lower verbal IQ and obesity vs. lower cognition and diabetes in the elderly. Nevertheless, our study describes different patterns of relationships of premorbid intelligence vs. cognitive impairment to BMI, insulin and diabetes in the elderly.

## Figures and Tables

**Table 1:** Demographic and metabolic status of the homebound elderly.

BMI Groups	BMI < 25 N = 67	25 < BMI < 30 N = 70	30 < BMI < 35 N = 67	BMI > 35 N = 83

Age, year (mean ± SD)*	80.2 ± 9.0	76.7 ± 8.4	77.5 ± 7.8	72.0 ± 6.7

**Female, No. (%)**	52 (78%)	47 (67%)	52 (78%)	72 (87%)

**Education N (%)**				
**0–8 years**	5 (13%)	9 (24%)	9 (24%)	15 (39%)
**9–12 years**	38 (22%)	41 (24%)	43 (25%)	50 (29%)
**> 12 years**	22 (30%)	20 (27%)	14 (19%)	18 (24%)

BMI, kg/m^2^ (Mean ± SD)*	22.4 ± 2.1	27.5 ± 1.4	32.4 ± 1.4	42.1 ± 6.9

Insulin, pmol/l (Mean ± SD)*	50.4 ± 37.6	76.9 ± 56.4	134.5 ± 245.7	154.1 ± 102.1

Diabetes, N (%)*	8 (12%)	21 (30%)	31 (46%)	50 (60%)

**Cardiovascular disease, N (%)**	27 (44%)	31 (45%)	25 (40%)	42 (51%)

Hypertension, N (%)**	55 (82%)	63 (90%)	64 (98%)	80 (96%)

**Stroke history, N (%)**	14 (21%)	17 (24%)	16 (24%)	16 (19%)

**ApoE 4, N (%)**	10 (17%)	16 (26%)	9 (15%)	24 (32%)

* Statistically significant, P < 0.0001

** Statistically significant, P = 0.002

**Table 2:** BMI, Verbal IQ and current cognitive status of the homebound elderly.

BMI Groups	BMI < 25 N = 67	25 < BMI < 30 N = 70	30 < BMI < 35 N = 67	BMI > 35 N = 83	P value
**NAART (Mean ± SD)**	101 ± 11	98 ± 12	96 ± 11	93 ± 11	0.0003
**MMSE (Mean ± SD)**	25.4 ± 4.2	25.3 ± 3.4	25.1 ± 3.6	25.4 ± 3.2	0.9140

**Table 3:** The univariate regression analysis.

	NAART ^1^Score		MMSE^2^ Score	
	β Estimate (SE)	P value	β Estimate (SE)	P value
**Age, year**	± 0.08 (0.08)	0.31	− 0.09 (0.02)	0.02
**BMI, kg/m^2^**	− 0.28 (0.08)	<0.001	± 0.03 (0.02)	0.27
**Insulin, pmol/l**	− 0.02 (0.01)	0.02	± 0.003 (0.22)	0.22
**Diabetes**	−3.81 (1.36)	<0.01	− 1.03 (0.43)	0.02
**Education^3^**				
**0–8 years**	− 16.53 (1.97)	<0.001	− 3.73 (0.65)	<0.001
**9–12 years**	− 8.15 (1.38)	<0.001	− 0.95 (0.45)	0.04
**> 12 years**	0	0	0	0

NAART: the North American Adult Reading Test MMSE: the Mini-Mental State Exam

Reference group for education is >12 years category

**Table 4:** The multivariate regression analysis.

	NAART^1^ Score		MMSE^2^ Score	
	β Estimate (SE)	P value	β Estimate (SE)	P value
**Age, year**	± 0.07 (0.09)	0.40	− 0.07 (0.03)	0.007
**BMI, kg/m^2^**	− 1.10 (0.45)	0.02	− 0.19 (0.14)	0.17
**BMI^2^**	± 0.01 (0.01)	0.02	± 0.003 (0.002)	0.14
**Insulin, pmol/l**	− 0.01 (0.01)	0.15	± 0.003 (0.003)	0.32
**Diabetes**	− 1.62 (1.50)	0.46	− 1.56 (0.43)	<0.001
**Education^3^**				
**0–8 years**	− 17.52 (2.24)	<0.001	− 3.95 (0.69)	<0.0001
**9–12 years**	− 8.99 (1.58)	<0.001	− 0.10 (0.49)	0.83
**> 12 years**	0	0	0	0

The models were adjusted for cardiovascular disease, hypertension, stroke and ApoE4. All of their associations were not statistically significant.

NAART: the North American Adult Reading Test MMSE: the Mini-Mental State Exam

Reference group for education is >12 years category

## References

[1] Elias PK, Elias MF, D’Agostino RB, Cupples LA, Wilson PW (1997). NIDDM and blood pressure as risk factors for poor cognitive performance. The Framingham Study. Diabetes Care.

[2] Gregg EW, Yaffe K, Cauley JA, Rolka DB, Blackwell TL (2000). Is diabetes associated with cognitive impairment and cognitive decline among older women? Study of Osteoporotic Fractures Research Group. Arch Intern Med.

[3] Grodstein F, Chen J, Wilson RS, Manson JE, Nurses’ Health Study (2001). Type 2 diabetes and cognitive function in community-dwelling elderly women. Diabetes Care.

[4] Lowe LP, Tranel D, Wallace RB, Welty TK (1994). Type II diabetes and cognitive function. A population-based study of Native Americans. Diabetes Care.

[5] Sinclair AJ, Girling AJ, Bayer AJ (2000). Cognitive dysfunction in older subjects with diabetes mellitus: impact on diabetes self-management and use of care services. All Wales Research into Elderly (AWARE) Study. Diabetes Res Clin Pract.

[6] Wu JH, Haan MN, Liang J, Ghosh D, Gonzalez HM (2003). Impact of diabetes on cognitive function among older Latinos: a population-based cohort study. J Clin Epidemiol.

[7] Peila R, Rodriguez BL, Launer LJ (2002). Type 2 diabetes, APOE gene, and the risk for dementia and related pathologies: The Honolulu-Asia Aging Study. Diabetes.

[8] Messier C, Awad N, Gagnon M (2004). The relationships between atherosclerosis, heart disease, type 2 diabetes and dementia. Neurol Res.

[9] Craft S, Asthana S, Schellenberg G, Cherrier M, Baker LD (1999). Insulin metabolism in Alzheimer’s disease differs according to apolipoprotein E genotype and gender. Neuroendocrinology.

[10] Luchsinger JA, Tang MX, Shea S, Mayeux R (2004). Hyperinsulinemia and risk of Alzheimer disease. Neurology.

[11] Klein S, Sheard NF, Pi-Sunyer X, Daly A, Wylie-Rosett J (2004). Weight management through lifestyle modification for the prevention and management of type 2 diabetes: rationale and strategies: a statement of the American Diabetes Association, the North American Association for the Study of Obesity, and the American Society for Clinical Nutrition. Diabetes Care.

[12] Fein G, Di Sclafani V (2004). Cerebral reserve capacity: implications for alcohol and drug abuse. Alcohol.

[13] Elias MF, Elias PK, Sullivan LM, Wolf PA, D’Agostino RB (2003). Lower cognitive function in the presence of obesity and hypertension: the Framingham heart study. Int J Obes Relat Metab Disord.

[14] Katzman R, Terry R, DeTeresa R, Brown T, Davies P (1988). Clinical, pathological, and neurochemical changes in dementia: a subgroup with preserved mental status and numerous neocortical plaques. Ann Neurol.

[15] Bright P, Jaldow E, Kopelman MD (2002). The National Adult Reading Test as a measure of premorbid intelligence: a comparison with estimates derived from demographic variables. J Int Neuropsychol Soc.

[16] Folstein MF, Folstein SE, McHugh PR (1957). “Mini-mental state”. A practical method for grading the cognitive state of patients for the clinician. J Psychiatr Res.

[17] Barnes DE, Tager IB, Satariano WA, Yaffe K (2004). The relationship between literacy and cognition in well-educated elders. J Gerontol A Biol Sci Med Sci.

[18] Haan MN, Mungas DM, Gonzalez HM, Ortiz TA, Acharya A (2003). Prevalence of dementia in older latinos: the influence of type 2 diabetes mellitus, stroke and genetic factors. J Am Geriatr Soc.

[19] Friend KB, Grattan L (1998). Use of the North American Adult Reading Test to estimate premorbid intellectual function in patients with multiple sclerosis. J Clin Exp Neuropsychol.

[20] Teasdale TW, Sørensen T, Stunkard AJ (1992). Intelligence and educational level in relation to body mass index of adult males. Hum Biol.

[21] Sørensen T, Sonne-Holm S, Christensen U, Kreiner S (1982). Reduced intellectual performance in extreme overweight. Hum Biol.

[22] Sørensen T, Sonne-Holm S (1985). Intelligence test performance in obesity in relation to educational attainment and parental social class. J Biosoc Sci.

[23] Li X (1995). A study of intelligence and personality in children with simple obesity. Int J Obes Relat Metab Disord.

[24] Halkjaer J, Holst C, Sørensen TI (2003). Intelligence test score and educational level in relation to BMI changes and obesity. Obes Res.

[25] Stolk RP, Breteler MM, Ott A, Pols HA, Lamberts SW (1997). Insulin and cognitive function in an elderly population. The Rotterdam Study. Diabetes Care.

[26] Vanhanen M, Koivisto K, Kuusisto J, Mykkänen L, Helkala EL (1998). Cognitive function in an elderly population with persistent impaired glucose tolerance. Diabetes Care.

[27] Hove O1 (2004). Weight survey on adult persons with mental retardation living in the community. Res Dev Disabil.

[28] Bhargava A, Fox-Kean M (2003). The effects of maternal education versus cognitive test scores on child nutrition in Kenya. Econ Hum Biol.

[29] Crnic KA, Sulzbacher S, Snow J, Holm VA (1980). Preventing mental retardation associated with gross obesity in the Prader-Willi syndrome. Pediatrics.

